# Author reply: Is the Effectiveness of Self-Visualization During Flexible Cystoscopy Gender-Dependent in Patients with no Previous Cystoscopy History? A Prospective Randomized Study

**DOI:** 10.1590/S1677-5538.IBJU.2025.0118

**Published:** 2025-03-29

**Authors:** Nurullah Hamidi

**Affiliations:** 1 University of Health Science Dr. Abdurrahman Yurtaslan Department of Urology Ankara Oncology Training and Research Hospital Ankara Turkey Department of Urology, University of Health Science Dr. Abdurrahman Yurtaslan, Ankara Oncology Training and Research Hospital, Ankara, Turkey

To the editor,

We appreciate the insightful comments and constructive feedback provided regarding our study (1), "Is the Effectiveness of Self-Visualization During Flexible Cystoscopy Gender-Dependent in Patients with no Previous Cystoscopy History? A Prospective Randomized Study." We welcome the opportunity to address the points raised and further clarify aspects of our methodology and findings.

Firstly, we acknowledge the concern regarding potential gender bias. One of the primary objectives of our study was to explore gender-based differences in pain perception and anxiety levels during flexible cystoscopy (FC). We believed that male and female patients experience procedural pain differently due to anatomical and psychological factors. Urethral length is short in females, making them less likely to experience pain during insertion of the cystoscope. Moreover, elongation in the craniocaudal diameter due to prostate enlargement, particularly in older men, and narrowing at the membranous urethra level may cause increased pain. However, we agree that a more detailed analysis of psychological variables such as baseline anxiety levels and pain tolerance could provide further insights into these differences.

Regarding the randomization strategy, our aim was to maintain an equal gender distribution within the SV and non-SV groups. The sequential assignment ensured a balanced male-to-female ratio, preventing inadvertent gender imbalances. While additional randomization techniques could potentially mitigate confounding factors, we believe our approach provided a fair and representative distribution of patients.

With respect to statistical power, our sample size was determined based on prior literature and power analysis calculations, ensuring sufficient strength to detect meaningful differences in both genders separately. Furthermore, our study (1) includes the largest number of female patients among previously published studies (2, 3). We agree that future studies with larger female cohorts could provide more conclusive evidence in this subgroup.

The suggestion to incorporate multivariate analysis to adjust for confounding factors such as prior medical procedures and anxiety disorders is an excellent point. While we excluded patients with prior cystoscopy experiences to ensure a uniform baseline, we recognize that other medical and psychological factors might have influenced patient responses. A future study incorporating a more extensive assessment of patient history and anxiety predisposition would add further depth to these findings.

We also appreciate the recommendation for longitudinal research to assess the long-term effects of SV on patient experience. While our study provides important insights into immediate procedural outcomes, future investigations could evaluate whether repeated exposure to SV fosters sustained reductions in anxiety and pain perception over multiple cystoscopy sessions. Additionally, exploring SV in combination with other pain management strategies, such as music therapy or relaxation techniques, could offer a more comprehensive approach to improving patient comfort.

Finally, we recognize the potential advantages of a blinded study design in reducing bias in outcome assessment, particularly for subjective measures like pain and satisfaction. However, as SV inherently involves real-time visualization, complete blinding may be challenging. Nevertheless, employing observer-blinded assessments or objective physiological stress markers could enhance the robustness of future studies.

We sincerely appreciate the thoughtful critique and suggestions, as they provide valuable directions for future research. We hope our clarifications address the concerns raised, and we look forward to continued discussions on optimizing patient comfort during cystoscopy procedures.

The Author
